# Monitoring moisture content in parchment coffee beans during drying using Fourier Transform near infrared (FT-NIR) spectroscopy: A dataset for calibrating chemometric-based models for moisture prediction

**DOI:** 10.1016/j.dib.2025.111436

**Published:** 2025-02-28

**Authors:** Sandrith Ordoñez-Lozano, Gentil A. Collazos-Escobar, Andrés F. Bahamón-Monje, Nelson Gutiérrez-Guzmán

**Affiliations:** a Centro Surcolombiano de Investigación en Café (CESURCAFÉ), Departamento de Ingeniería Agrícola, Universidad Surcolombiana, Neiva-Huila, Colombia; b Grupo de Análisis y Simulación de Procesos Agroalimentarios (ASPA), Instituto Universitario de Ingeniería de Alimentos–FoodUPV, Universitat Politècnica de València, Camí de Vera s/n, Edificio 3F, 46022 Valencia, Spain; c Departamento de Ingeniería Agroindustrial, Facultad de Ingeniería, Universidad Surcolombiana, Neiva, Huila, Colombia

**Keywords:** Monitoring, Quality control, Process simulation, Coffee post-harvest, Chemometrics

## Abstract

Maintaining the quality of coffee across each stage of the coffee value chain is critical, with proper bean drying being essential for preserving product shelf life and moisture stability. This work compiles a dataset collected during the mechanical drying process of parchment coffee beans, monitoring moisture content alongside their corresponding near-infrared (NIR) spectra. The aim was to evaluate the application of NIR spectroscopy for predicting moisture content during drying, leveraging NIR as a reliable, rapid, and non-destructive technology for routine monitoring of the coffee drying process. Drying kinetics of parchment coffee beans were determined using a mechanical coffee dryer, with moisture content gravimetrically monitored at various drying times. At each drying point, NIR spectra were acquired using a Spectrum Two N FT-NIR Spectrometer equipped with a high-resolution Indium Gallium Arsenide (InGaAs) detector, operating in diffuse reflectance mode. NIR spectra were collected over a wavelength range of 4000–12000 cm⁻¹ (830–2500 nm), with a 4 cm⁻¹ interval, 8 cm⁻¹ resolution, and 64 scans. This work explored moisture content from fresh coffee (52 % wet basis; w.b.) to 8 % w.b., examining spectral changes throughout the entire drying process. The compiled dataset includes experimental drying kinetics and FT-NIR spectra in Excel format, organized according to experimental conditions. This dataset provides a valuable foundation for further analysis and for calibrating predictive models of moisture content during coffee drying, highlighting the high potential of NIR spectroscopy for industrial-scale drying control and monitoring in the coffee industry.

## Graphical abstract

**Figure:**
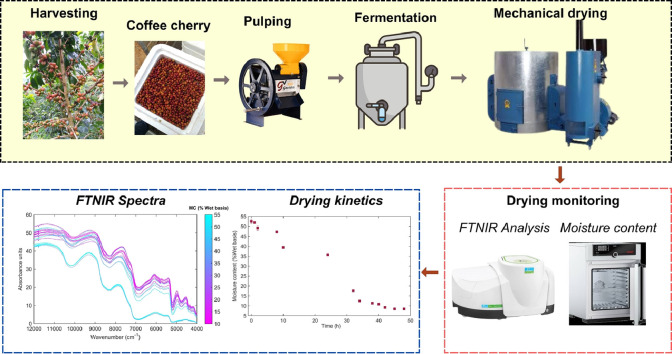

Specifications Table

**Table:** 

Subject	Food Science
Specific subject area	Food technology, Food engineering.
Type of data	Excel files: Moisture of parchment coffee and Near-infrared spectral data
Data collection	Moisture of parchment coffee (Wet basis), Fourier transform near infrared spectra (FT-NIR).
Data source location	The experimental dataset presented in this work belongs to Centro Surcolombiano de Investigación en Café (CESURCAFÉ) from the Universidad Surcolombiana, Neiva-Huila, Colombia.
Data accessibility	Repository name: Mendeley Data Data identification number: DOI: 10.17632/7c6gx7rr6d.1 Direct URL to data: https://data.mendeley.com/datasets/7c6gx7rr6d/1

## 1 Value of the Data

• This data establishes a basis for optimizing coffee quality by monitoring moisture levels during the drying process, ensuring the drying process for optimal preservation/development of flavor and aroma.
• This data facilitates the development of predictive models that enable monitoring and control of the drying process, helping achieve consistent quality across different coffee batches.
• This data enhances process efficiency by enabling real-time adjustments based on continuous moisture monitoring, thereby optimizing resource use in coffee drying operations.
• This data supports defect prevention by allowing for early detection of moisture changes, enabling immediate corrective actions to maintain product integrity.
• This data provides insights into the physicochemical properties of coffee through FT-NIR analysis, informing production and marketing strategies for improved market positioning.

## 2 Background

Quality is a fundamental factor in the coffee industry's economic landscape, with precise drying techniques playing a crucial role in preserving product shelf life during both storage and transportation. Moisture content in coffee beans is a key variable that requires careful control to prevent quality degradation, which can result in discoloration, undesirable flavors, and the growth of mold and other microorganisms that compromise quality [[1], [2], [3]]. FT-NIR technology is an effective, complementary analytical method that can be routinely employed to predict coffee properties, such as moisture content [4]. This vibrational spectroscopy technique allows for a detailed assessment of food chemical composition and identification of specific spectral features of chemical compounds [5] providing a basis for developing robust predictive models. This work aims to streamline processes in large-scale coffee industries by applying NIR sensors to accurately predict moisture content, using a clean, non-destructive approach with near-infrared wavelengths ranging from 780 to 2500 nm [2]. While most prior studies have focused on moisture content prediction in green and roasted beans, Colombia a major coffee-producing country dries its beans in parchment form, highlighting the relevance of this data for real-time moisture monitoring using NIR technology.

## 3 Data Description

The experimental data were summarized in two excel files, which are described below.

**Fig. 1:**
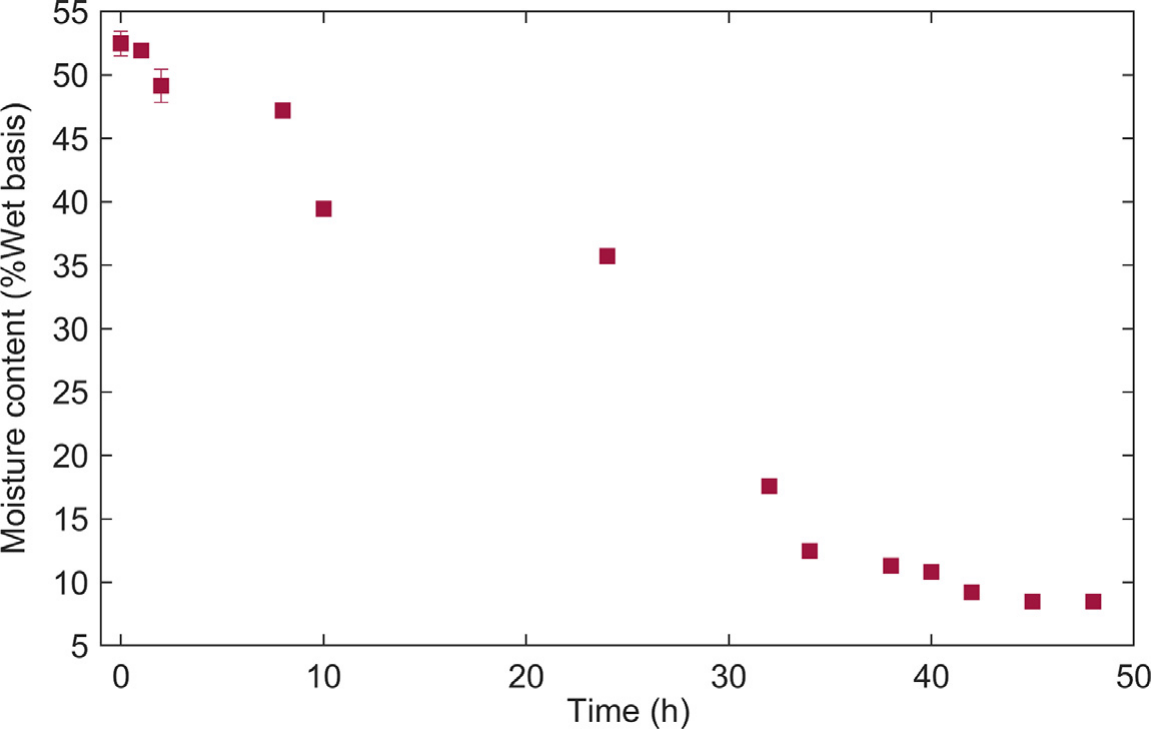
Drying kinetics of coffee samples. Moisture content of coffee beans is expressed as mean ± standard deviation at each point of the drying process.

**Moisture_values:** The collected dataset encompasses variations in the moisture content of dried parchment coffee beans, which were processed using the wet method and determined gravimetrically according to Colombian technical standard NTC 2325. The results are expressed as a percentage of moisture on a wet basis. Measurements cover a broad range, from an initial moisture content of 52 % post-depulping to a final value of 8 %, which is two percentage points below the optimal range of 10–12 % for long-term storage. This extensive variability in moisture data was employed to enhance the quality and robustness of the predictive model developed using FT-NIR. Columns 3 and 4 of the dataset present the standard deviation and coefficient of variation, respectively, of the three replicates taken at various time intervals during the drying process. Fig. 1 clearly illustrates the decreasing trend in moisture content over time (h), demonstrating the effectiveness of the drying process in reducing the water content of the coffee beans.

**Fig. 2:**
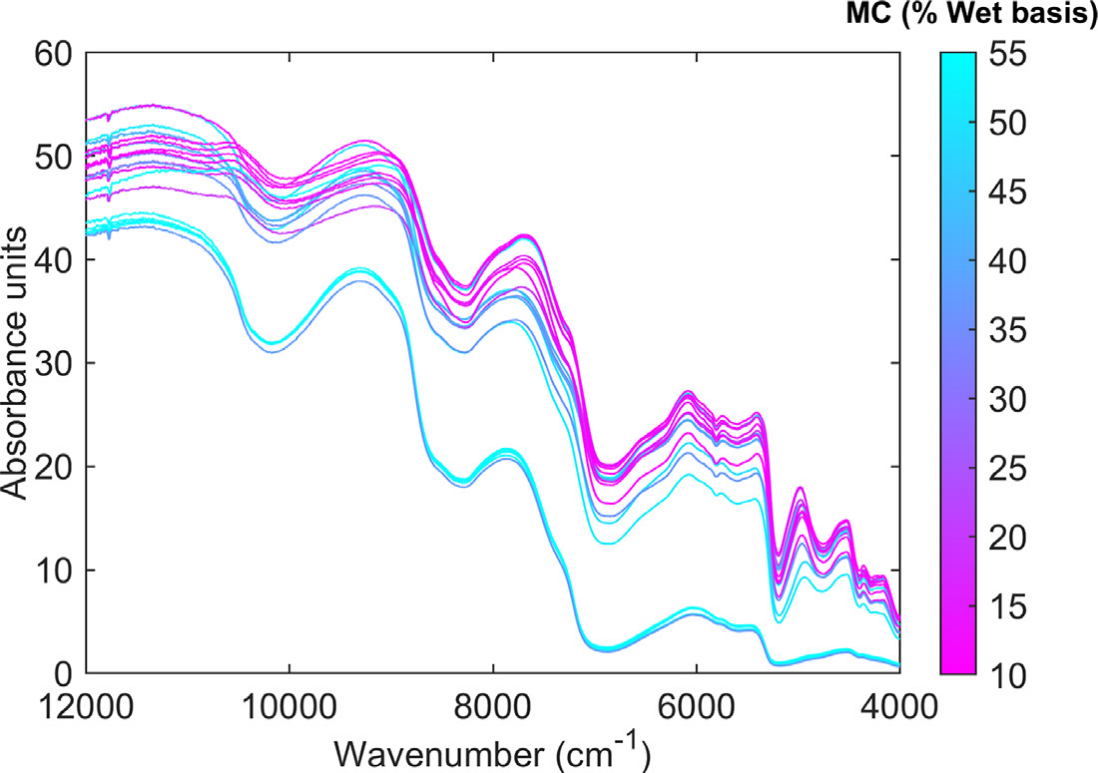
Average Fourier Transform near infrared spectra of coffee beans at different moisture levels during the drying process.

**FTNIR_data** The spectral dataset was acquired and recorded in the near-infrared region as absorbance spectra within a wavelength range of 4000–12000 cm⁻¹ (Fig. 2) using Spectrum IR software (Version 10, PerkinElmer, USA). These spectra were obtained from dried parchment coffee samples undergoing the drying process and contain information about the physical and chemical properties of the beans. Of particular interest in this work is the parameter of “moisture content present in the beans.” Through calibration and the application of chemometric-based techniques, a predictive model could be developed for monitoring this parameter during drying. The data indicate that the first column of the file represents the wavenumber (cm⁻¹) for all infrared spectra, while columns 2 to 77 contain the absorbance values for each sample and their respective replicates.

## 4 Experimental Design, Materials and Methods

**Fig. 3:**
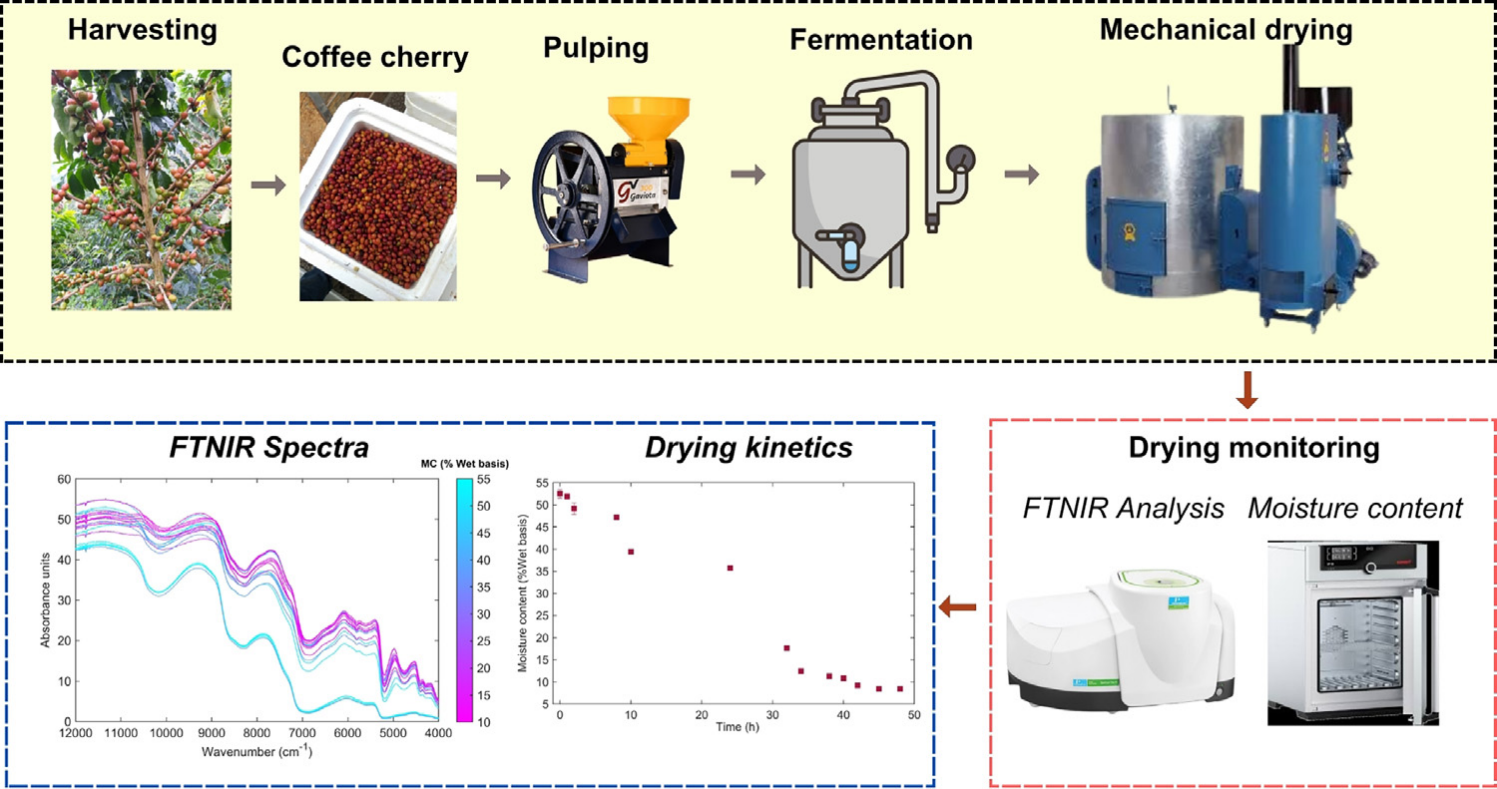
Experimental procedure for monitoring the drying kinetics of coffee beans using Fourier Transform Near Infrared (FT-NIR) spectroscopy.

Cherry coffee samples of Castillo, Caturra, Colombia, and pink Bourbon varieties (*Coffee arabica* L.) from different growing areas in the Huila-Region of Colombia, were collected. Wet processing was applied to samples at the pilot plant of the Centro Surcolombiano de Investigación en Café (CESURCAFÉ) in Neiva-Huila, Colombia. After depulping, the coffee samples were subjected to a fermentation process for 24 h, followed by washing and mechanical drying in an Ingesec equipment (Reference, INGESEC, Colombia) set to a temperature of 40 °C, a frequency of 36.6 Hz, and agitation every hour for 35 s until an optimal moisture range of 10–12 % w.b. was achieved (Fig. 3).

During the drying process for each coffee variety, approximately 30 g of representative sample were taken from each batch in the drying point. A stabilization period of approximately 10 min was allowed prior to measurements to ensure uniform moisture conditions in the beans. Subsequently, the moisture content in the coffee beans was measured using the gravimetric method [6]. For this, samples of 5 g were placed in an oven (UF55, Memmert GmbH + Co. KG, Schwabach, Germany) at a temperature of 105 ± 1 °C for approximately 24 h (Fig. 3). This process continued until a constant weight was achieved, indicating the removal of residual moisture. This methodology enabled a detailed and accurate analysis of the properties of each coffee variety in relation to its drying process.

For FT-NIR spectral acquisition, samples of 12 g of dry parchment coffee were placed in a petri dish, ensuring the removal of any external residues around the dish to prevent interference during measurements. The petri dish was then positioned in a Spectrum Two NIR spectrometer (PerkinElmer, Inc., USA), equipped with a rotational accessory designed to ensure representative scanning of the entire sample within the dish. Spectral data acquisition was conducted using diffuse reflectance mode, employing a high-resolution InGaAs detector. The detector operated within a wavenumber range of 12000–4000 cm⁻¹, with a data interval of 4 cm⁻¹ and a spectral resolution of 8 cm⁻¹. Each spectrum was obtained by averaging 64 consecutive scans to ensure precision and consistency. The final spectrum contained 2000 wavenumber data points. Prior to each series of measurements, the spectrometer was calibrated using a reference standard to correct the baseline and minimize instrumental drift. Additionally, environmental conditions such as relative humidity (25 ± 5 %) and temperature (22 ± 2 °C) were controlled and monitored to reduce their potential influence on spectral readings. To ensure data reliability, three replicates were performed for each sample, with each replicate measured at different points of the sample surface. Between replicates, the petri dish was rotated to account for sample variability. This methodology ensures the acquisition of high-quality spectral data, suitable for subsequent chemometric analysis and predictive modeling.

**Fig. 4:**
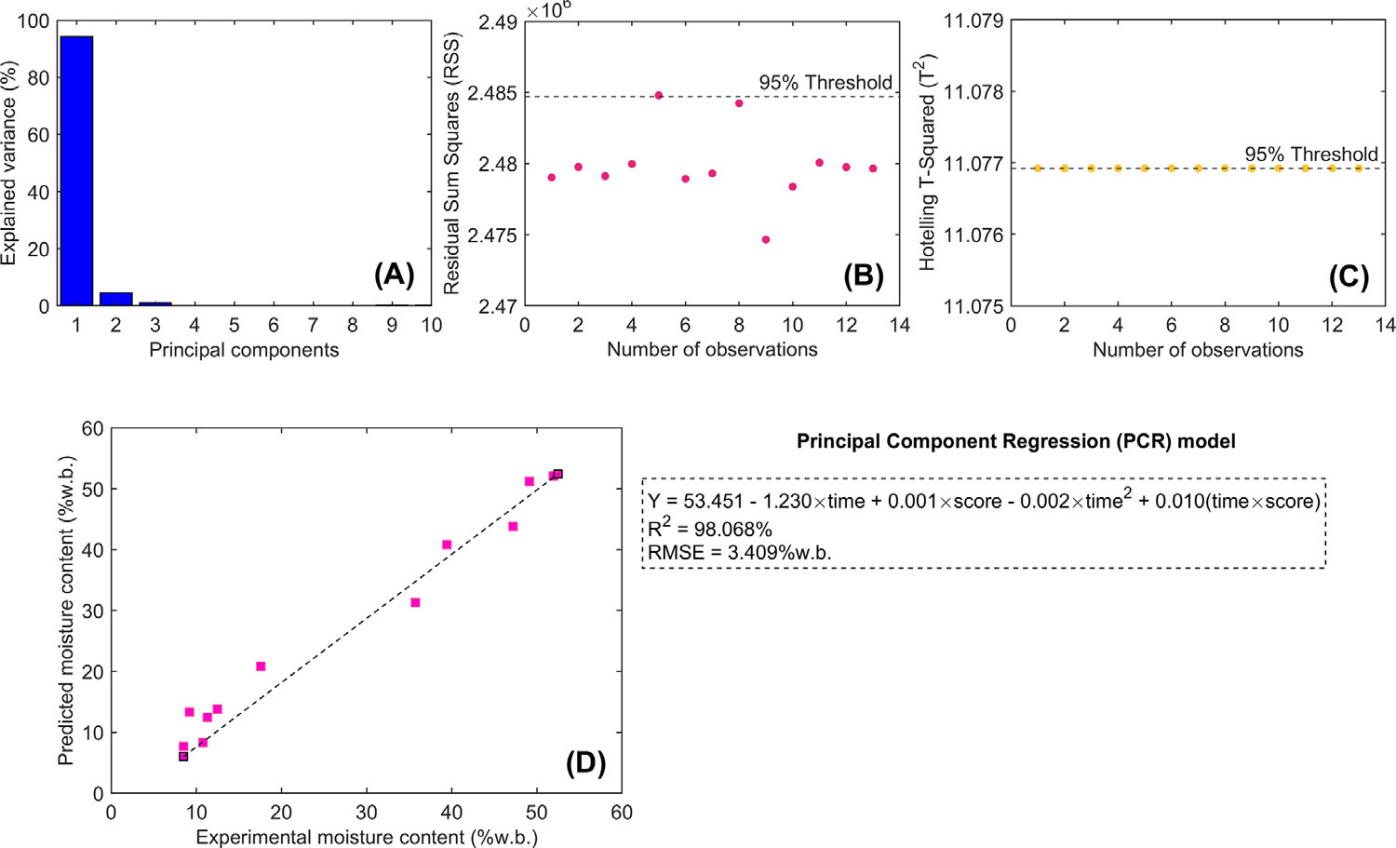
Example of a calibrated Principal Component Regression (PCR) model used to predict moisture content in coffee beans during drying, based on non-invasive Fourier Transform Near Infrared (FT-NIR) spectroscopy. The figure presents the statistical results of the PCR model, including: (A) explained spectral variance by principal components (PCs), (B) residual sum of squares (RSS), and (C) Hotelling's T-squared (T^2^) control charts for detecting outlier data. Additionally, it compares the experimental and predicted moisture content values obtained from the PCR model calibrated using FT-NIR spectral data.

An example of calibrating a chemometric-based model for moisture content prediction using non-invasive FT-NIR is illustrated in Fig. 4. To calibrate a chemometric-based model capable of predicting the moisture content of beans during the drying process, a Principal Component Regression (PCR) model was formulated (Eq. 1). This model is developed in two steps: first, a Principal Component Analysis (PCA) model is calibrated and validated. Then, the PCA scores (spectral observations projected onto the latent-structure eigen space coordinates of the PCA model), along with their corresponding drying times, serve as regressors in the calibration of a subsequent multivariate linear regression (MLR) model. For this purpose, spectral data (Fig. 2) were modeled using MATLAB® R2023a (The MathWorks Inc., Natick, MA, USA). The “pca” MATLAB function was used to compute the explained variance by principal components (PCs), PCA scores, PCA loadings, Residual Sum of Squares (RSS), and Hotelling's T-squared (T²) multivariate control statistics. These served as the basis for detecting outlier observations (RSS and T² > 95th percentile control limit; Fig. 4B and C), reducing the dimensionality of the spectral space, and summarizing spectral variability into a smaller number of latent variables [7]. Subsequently, the PCA scores corresponding to the first PC (score_pc1_) and drying time (Fig. 1) were used as regressors in a classical MLR model [8] (an MLR model calibrated using PCA scores as regressors is often known as a PCR model).

(1) $$Y=b_{0}+b_{1}\text{time}+b_{2}\text{scor}e_{\text{pc}1}+b_{3}\text{tim}e^{2}+b_{4}\left(\text{scor}e_{\text{pc}1}\times \;\text{time}\right)$$

The score_pc1_ was selected due to it represented 94.22 % (Fig. 4A) of the spectral variance captured in a single latent variable. Nonetheless, determining the optimal number of PCs for the PCR model should be considered an additional step in optimizing a chemometric-based predictive model.

The PCR model fitting was performed using the “fitlm” MATLAB function, which finds the optimal values of the PCR model parameters (b_i_) by minimizing the mean square error (MSE, Eq. 2) between the experimental moisture content (Y) and the predicted values (Y_pred_). Additionally, the predictive power of this mathematical model was evaluated using the coefficient of determination (R^2^; Eq. 3) and the root mean square error (RMSE; Eq. 4).

(2) $$\text{MSE}=\frac{\sum_{i=1}^{n}{\left(Y-Y_{\text{pred}}\right)}^{2}}{N}$$

(3) $$R^{2}\left(\%\right)=100-\frac{\sum_{i=1}^{N}{\left(Y-Y_{\text{pred}}\right)}^{2}}{\sum_{i=1}^{N}{\left(\bar{Y}-Y_{\text{pred}}\right)}^{2}}$$

(4) $$\text{RMSE}=\sqrt{\text{MSE}}$$

Where N is the number of experimental data points.

As a result, the PCR chemometric-based model allowed for an accurate prediction of moisture content (R^2^ > 98 % and RMSE < 3.4 %w.b; Fig. 4D) highlighting the potential of FT-NIR spectroscopy to support real-time industrial-scale drying processes and monitor moisture content in the coffee industry.

## Limitations

None.

## Ethics Statement

The dataset collected in this work did not involve human subjects, animal experiments, or any data collected from social media platforms.

## Credit author statement

**Sandrith Ordoñez-Lozano:** Methodology, Data curation, Writing. **Gentil A. Collazos-Escobar:** Conceptualization, Methodology, Software, Data curation, Visualization, Writing, Original draft preparation. **Andrés F. Bahamón-Monje:** Software, Data curation, Writing, Original draft preparation. **Nelson Gutiérrez-Guzmán:** Supervision, Writing- Reviewing and Editing.

## Data Availability

Mendeley DataFourier Transform Near Infrared (FT-NIR) spectral monitoring of parchment coffee beans during drying (Original data)
